# Policosanol composition, antioxidant and anti-arthritic activities of milk thistle (*Silybium marianum* L.) oil at different seed maturity stages

**DOI:** 10.1186/s12944-018-0682-z

**Published:** 2018-04-16

**Authors:** Saoussem Harrabi, Azza Ferchichi, Asma Bacheli, Hayet Fellah

**Affiliations:** Laboratory of Clinical Biochemistry, LR99ES11, Faculty of Medicine Tunis, 15 street Djebel Lakhdar, Rabta, 1007, University of Tunis El Manar, Tunis, Tunisia

**Keywords:** Milk thistle, Oil, Anti-arthritic activity, Antioxidant capacity, Policosanol, Maturity stage

## Abstract

**Background:**

Several anti-arthritic drugs and synthetic antioxidants have wide pharmaceutical uses and are often associated with various side effects on the human health. Dietary seed oils and their minor components like policosanol may offer an effective alternative treatment for arthritic and oxidative-stress related diseases. The biological effects of seed oils were affected by different parameters such as the stage of seed maturity. Hence, this study seeks to determine the policosanol content, antioxidant and anti-arthritic activities of milk thistle (*Silybium marianum* L.) oil extracted at various stages of seed maturation.

**Methods:**

Milk thistle oil samples were extracted from seeds collected at three maturation stages (immature, intermediate, and mature). The 2,2-diphenyl-1-picrylhydrazyl (DPPH) and 2,2′-azino-bis (3-ethyl-benzthiazoline-6-sulfonic acid) (ABTS) radical scavenging assays were used to determine the antioxidant activity of the extracted oils. The anti-arthritic activity of oil samples was evaluated with bovine serum protein denaturation and egg albumin denaturation methods. Gas chromatography coupled to mass spectrometry (GC-MS) was employed to determine the policosanol profile.

**Results:**

Policosanol profile, antioxidant and anti-arthritic activities of milk thistle oil were influenced by the seed maturity stages. The oil extracted from the immature seeds had the highest total policosanol content (987.68 mg/kg of oil) and displayed the maximum antiradical activity (96.42% and 90.35% for DPPH test and ABTS assay, respectively). Nine aliphatic alcohols were identified in the milk thistle oil. The dominant poliosanol in the mature seed oil was octacosanol (75.44%), while triacontanol was the major compound (40.25%) in the immature seed oil. Additionally, the maximum inhibition of bovine serum protein denaturation (92.53%) and egg albumin denaturation (86.36%) were observed in immature seed oil as compared to mature seed oil. A high correlation was found between the total policosanol content, anti-arthritic activity and antioxidant capacity of oil.

**Conclusions:**

The milk thistle oil exhibited a potential anti-arthritic and antioxidant activities and that it might contribute to the protection of humans from a variety of diseases like rheumatoid arthritis. Also, it could serve as natural antioxidant and anti-arthritic agents for application in the food industries and pharmaceutic. Policosanol level in the seed oils might contribute to their anti-arthritic and antioxidant activities.

## Background

Oxidative stress in the human tissues leads to several diseases like cancer, diabetes, arthritis, atherosclerosis and chronic inflammatory disorders. Nearly one-fifth of the world’s population is affected by rheumatoid arthritis [1]. The anti-arthritic and anti-inflammatory drugs presently used are characterized by their possible adverse effects on the body such as ulcers and cardiovascular problems [2, 3]. Also, the use of synthetic antioxidants like butylated hydroxyanisole and butylated hydroxytoluene in the pharmaceutical and food industries has various side effects [4, 5]. Therefore, much attention has been focused on the development of alternative anti- arthritic agents and antioxidants from natural resources. It was reported that the intake of natural antioxidants has been inversely associated with morbidity and mortality from degenerative disorders and other infections [6]. Medicinal plant may offer an alternative source for the anti-arthritic, anti-inflammatory and antioxidant drugs. The antioxidant capacity of plant extracts was influenced by different parameters such as environmental conditions, genotype and stage of maturity [7–10].

Policosanol is a mixture of long-chain (C20 to C36) aliphatic primary alcohols exhibiting various beneficial effects on the human health. It was originally isolated from sugar cane wax and is also found in a number of other natural sources such as beeswax and vegetable oils [11–13]. Policosanol may be effective in the treatment of hypercholesterolemia by inhibiting hepatic cholesterol biosynthesis, enhancing LDL catabolism and increasing high density lipoprotein levels in serum [14, 15]. These aliphatic alcohols have also been shown to possess anti-inflammatory and antioxidant effects [13, 16]. Octacosanol (C28-OH), the main active policosanol, has gained attention due to its health benefits including anti-parkinsonian, antinociceptive and anti-inflammatory effects [17, 18]. Triacontanol (C30-OH) is able to induce anti-inflammatory responses in animals, prevent oxidative stress and inhibit lipid peroxidation [19, 20]. Millán et al. [21] revealed that the nutraceutical combination containing policosanol, berberine, and red yeast rice induced significant improvements in plasma lipids. Therefore, there is growing interest in the identification of natural sources of policosanol for the functional foods and nutraceutical applications [22]. Contents and compositions of policosanol in different plant sources such as rice bran, wheat bran, sugar cane wax, corn kernel, green tea leaves, grain sorghum, perilla seeds and grape seed have been reported [12, 22–25].

Milk thistle (*Silybum marianum* L.) is an important medicinal plant from the family *Asteraceae*. The bioactive compounds are mainly concentrated in its seeds which have been used for more than 2000 years to treat liver diseases. Milk thistle seeds contain 17.5–30.5% of lipids rich in unsaturated fatty acids and 1–3% of silymarin [10, 26, 27].. Recently, certain *Silybum marianum* accessions have begun to be cultivated in several countries and the specie is undergoing domestication for making the supply of silymarin sustainable [28]. Previously published studies examined the triacylglycerol, fatty acid, tocopherol, sterol and polyhpenol composition of milk thistle seeds [10, 26, 29–32]. Significant differences were observed between milk thistle cultivars for the content of bioactive compounds [10]. Also, the antioxidant properties of ethanolic extracts of milk thistle seeds and methanolic extracts of cold-pressed milk thistle seed oil have been reported [10, 31].To the best of our knowledge, however, no data has been published on the policosanol composition and the anti-arthritic activity of milk thistle oil. Therefore, this study aimed to examine the policosanol profile, anti-arthritic and antioxidant activities of milk thistle oil at different stages of seed maturity. Such data could serve for the evaluation of nutritional and health impact of milk thistle oil and for the development of new source of natural bioactive compounds.

## Methods

### Materials and reagents

The milk thistle (*Silybium marianum* L.) seeds were collected from plants growing in region of Sousse (Centre of Tunisia), during April and June, 2012. The seeds were authenticated at the National Botanical Research Institute Tunisia (INRAT). Seeds were selected according to external color; green seeds were chosen as immature stage, mahogany brown seeds as the intermediate stage and dark brown seeds as the last stage of maturity (mature stage).

Chloroform, methanol and petroleum ether were purchased from Lab-Scan analytical Sciences (Poland). Ethanol, diethyl ether and n-hexane were obtained from Scientific Limited (Northampton, UK). 2, 7-Dichlorofluorescein, DPPH (2, 2-diphenyl-1-picrylhydrazyl radical) and the standard 1-eicosanol were purchased from Sigma-Aldrich, Co. (St. Louis, MO, USA). Potassium hydroxide pellets and anhydrous sodium sulfate were obtained from AppliChem (Darmstadt, Germany). ABTS, 2,29-azinobis(3-ethylbenzothiazoline-6- sulfonic acid) diammonium salt, and potassium persulfate (di-potassium peroxdisulfate) were obtained from Sigma-Aldrich (Poole, Dorset, UK).

### Seed oil extraction, saponification and thin layer chromatography

The oils were extracted by the method of Folch et al. [33]. Seeds (2.5 g) were washed with boiling water for 5 min and then crushed in a mortar with chloroform/methanol (2:1, *v*/v). The mixture was centrifuged at 3000 g for 15 min and the lower chloroformic phase containing the total lipids was kept and dried in a rotary evaporator at 40 °C.

The saponification and the Thin layer chromatography analysis of unsaponoifiables were done according to the previous reports [12]. The unsaponifiable fraction and the authentic 1-eicosanol were spotted on preparative silica gel thin-layer plates (silica gel 60G F254) and developed with hexane–diethyl ether (65:35, *v*/v). After development, the plate was sprayed with 2,7-dichlorofluorescein and viewed under UV light. The band corresponding to aliphatic alcohols was scraped, extracted three times with chloroform–diethyl ether (1:1, v/v), filtered to remove the residual silica, dried in a rotary evaporator and stored at − 10 °C.

### Analysis of policosanol by GC-MS

GC-MS analyses were performed using a capillary HP-5MS column (30 m × 0.25 mm I.D., 0.25 μm film thickness; Agilent Technologies) with gas chromatography (Agilent Technologies 7820A) coupled directly to the mass detector (Agilent Technologies 5975 series MSD). Helium was used as carrier gas, with a constant flow rate of 1 ml/ min. The injector and detector temperatures were 230 °C. The oven temperature was programmed from 150 to 320 °C at 10 °C·min − 1 from 150 to 250 °C and at 5 °C·min − 1 from 250 to 320 °C. Manual injection of 1 μL of the aliphatic alcohol solution was performed in the split mode at a 10:1 split ratio. The policosanol compounds were identified by comparing their mass spectra with the Wiley 275.L Mass Spectral Library.

### Measurement of in vitro antioxidant activity

#### DPPH test

The antioxidant activity of seed oil samples was determined using DPPH radicals as described by Kozłowska et al. [34]. Fifty mg of oil was dissolved in 3 mL of ethyl acetate and then 1 mL of oil solution was diluted with 2.75 mL ethyl acetate. 0.25 mL of DPPH solution (1 mM) was added and the mixture was shaken vigorously for 10s in a vortex apparatus. After 20 min, the absorbance was measured at 515 nm using UV/Vis scanning spectrophotometer (Model 2650, Labomed, Inc. U.S.A) and the percent of inhibition was calculated using this formula:

Inhibition (%) = {(Absorbance of the control – Absorbance of sample)/Absorbance of the control}/ × 100.

#### ABTS antioxidant assay

The ABTS radical scavenging capacity of the oil was measured using the method described by Rubalya and Neelamegam [35]. The ABTS^+^ radical was generated by oxidation of 2.5 ml of ABTS solution (7 mM) with potassium persulfate (14.7 mM). The mixture is kept in the dark for 16 h at room temperature (25 °C). Before usage, the mixture was diluted with water to obtain an absorbance of 0.70 ± 0.05 at 734 nm. The radical scavenging activity is assessed by mixing 2 ml of this diluted ABTS+ solution with different oil samples dissolved in benzene. After 30 min, the percentage inhibition at 734 nm was calculated for each sample relative to blank absorbance. The percentage inhibition of ABTS radical by the oils was calculated using the equation described in the DPPH assay.

### Measurement of in vitro anti-arthritic activity

In vitro anti-arthritic activity was evaluated using bovine serum protein denaturation method and egg albumin denaturation method [36] with some modifications [37].

#### Bovine serum albumin (BSA) denaturation method

Test solution (0.5 ml) consisted of 0.45 ml bovine serum albumin (0.5% *w*/*v* aqueous solution) and 0.05 ml of oil samples. Then the samples were incubated at 37 °C for 20 min followed by incubation at 57 °C for 3 min. After cooling the samples, 2.5 ml phosphate buffer (pH 6.3) was added to each tube. UV-Visible spectrophotometer was used to measure the absorbance at 660 nm. The control represents 100% protein denaturation. For test control solution (0.5 ml) 0.05 ml distilled water was used instead of oil sample while for product control (0.5 ml) 0.45 ml distilled water and test solution (0.05 ml) were used. The percentage inhibition of protein denaturation was calculated by the following formula:

Percentage inhibition = {(Absorbance of test solution- Absorbance of the control)/Absorbance of the control}/ × 100.

#### Egg albumin denaturation method

The reaction mixture (5 ml) was comprised of 0.2 ml of fresh hen’s egg albumin, 2.8 ml of phosphate buffered saline (PBS, pH 6.4) and 2mlof oil samples. Similar volume of double distilled water served as control. Then the mixture was incubated at 37 °C in the incubator for 15 min and then heated at 7°0 C for 5 min. After cooling, their absorbance was measured at 660 nm by using pure blank. The percentage inhibition of protein denaturation was calculated as mentioned in Bovine serum albumin (BSA) denaturation method.

### Statistical analysis

The analysis of the studied samples was performed in triplicate and the results were expressed as means± standard deviation (SD). Statistical analysis was performed by using the Proc ANOVA in SAS (Software version 8). Duncan’s Multiple Range Test was applied. The Pearson correlation coefficient (r) was used to examine the relation between the main parameters.

## Results and discussion

### Total policosanol content

To the best of our knowledge, the policosanol composition of milk thistle oil has never been previously reported. As seen in Table 1, the total policosanol content of the mature milk thistle seeds was 574.4 mg/kg of oil (equivalent to about 175.2 mg/kg of dry weight) and was higher than those of grape seed and rice bran oils (171.17–245.15 mg/kg of oil) [23]. However, it was lower than those of other plant sources such as sorghum kernel (800 mg/kg dry weight) and green tea leaves (726.2–1363.6 mg/kg dry weight) [22, 24]. Milk thistle seed oil had high content of policosanols which are considered important from a nutritional and functional point of view. Policosanol contents in vegetables are shown to vary due to many factors like species, ripening grade of fruits and type of tissue [12, 23, 38].

**Table 1:** Total policosanol content and antioxidant capacity of milk thistle oil, at three seed maturity stages

Maturation stage	Policosanol content (mg/kg oil)	DPPH^a^ scaveninig ability	ATBS^b^ scaveninig ability
Immature	987.68 ± 16.41	96.42 ± 2.28	90.35 ± 2.6
Intermediate Mature	612.24 ± 5.89	84.46 ± 1.52	77.48 ± 1.8
	574.49 ± 6.34	76.52 ± 1.76	70.25 ± 1.34

Values were expressed as means ± SD of triplicate experiments

^a^*DPPH* 1,1-diphenyl-2-picrylhydrazyl radical and results are expressed as percentage of inhibition of DPPH by the oil

^b^ABTS : 2,2′-azino-bis (3-ethyl-benzthiazoline-6-sulfonic acid) and results are expressed as percentage of inhibition of ABTS by the oil

The content of policosanol in milk thistle oil was affected by seed maturity stages (Table 1). It was higher in the immature seeds (987.68 mg/kg oil) and then decreased during seed maturation. Also, a decreasing trend was observed for the policosanol content during the early stages of corn kernel development [12]. In contrast, during *Tilia tomentosa* leaf development, total primary alcohol content was found to increase from 0.031% to 0.197% of dry weight [38]. These quantitative differences observed in the policosanol content during seed development could be linked to change in the activity of fatty acyl-coA reductase which converts fatty acyl-coA into fatty alcohol.

### Policosanol composition

The aliphatic alcohols fraction of the milk thistle oil was submitted to the GC-MS analysis. Nine aliphatic alcohols were identified in the oil samples and they range from C_22_ to C_32_ (Table 2). The dominant policosanols in the mature seed oil were Octacosanol (75.44%) and triacontanol (8.61%), however the other aliphatic alcohols detected including C_22_, C_23_, C_24_, C_26_, C_27_, C_29_, and C_32_ were present in amounts ranging from 1.52 to 3.34%. Milk thistle oil extracted from mature seeds could serve as natural source of octacosanol, which has been shown to exhibit various beneficial effects such as anti-parkinsonian, antinociceptive and anti-inflammatory effects [17, 18]. Octacosanol is also the single most abundant policosanol in sugar cane wax (60–70%) and perilla seeds (55.93%) with a minor quantity of many other policosanols [23, 25]. The policosanol composition in vegetable oils was greatly source dependent [23]. In the commercial green tea leaves, octacosanol (29.9–42.7%) and triacontanol (27.4–41.5%) were the main policosanol components [22]. Olive oil was characterized by the predominance of hexacosanol, tetracosanol and octacosanol [23]. Harrabi et al. [12] reported that dotriacontanol (30.1–35.5%) was the major policosanol in whole corn kernel, followed by triacontanol (17.7–24.8%) and tetracosanol (15.2–25.7%). However in wheat, tetracosanol was the most abundant compound, followed by docosanol and hexacosanol [39].

**Table 2:** Policosanol composition of milk thistle oil extracted from mature seeds

Policosanol (Chemical formula)	Percent
Docosan-1-ol (C_22_-OH)	1.52 ± 0.24
Tricosan-1-ol (C_23_-OH)	2.12 ± 0.39
Tetracosan-1-ol (C_24_-OH)	2.02 ± 0.50
Hexacosan-1-ol (C_26_ -OH)	2.50 ± 0.16
Heptacosan-1-ol (C_27−_OH)	1.90 ± 0.25
Octacosan-1-ol (C_28_-OH)	75.44 ± 2.41
Nonacosan-1-ol (C_29_-OH)	2.55 ± 0.62
Triacontan-1-ol (C_30_-OH)	8.61 ± 1.20
Dotriacontan-1-ol (C_32_-OH)	3.34 ± 0.56

Values were expressed as means ± SD of triplicate experiments

**Fig. 1:**
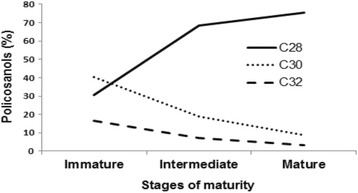
Changes in octacosanol, triacontanol and dotriacontanol levels in milk thistle oil during seed maturation

Figure 1 shows that the percentages of octacosanol, triacontanol and dotriacontanol were largely influenced by seed maturity stages. In immature seeds, the dominant alcohol was C_30_ (40.25%), followed by C_28_ (30.42%) and C_32_ (16.36%). During seed maturation, the percentage of octacosanol increased rapidly, while those of triacontanol and dotriacontanol decreased. The levels of the other detected compounds were relatively constant, as the seed developed. In agreement to our present trends, the level of triacontanol was also found to decrease during corn kernel development [12]. Immature seeds are rich in triacontanol which is essential for their development. In fact, triacontanol has been reported to stimulate plant growth, increase dry weight, prevent oxidative stress and act as an inhibitor of lipid peroxidation [20, 40]. The variation in the policosanol composition might be due to the physiological changes that accompany ripening of seeds. Triacontanol exhibited anti-inflammatory action in animals and has been suggested to be an effective anti-inflammatory drug [19]. Thus, immature milk thistle seeds could be exploited as a natural source of this bioactive compound.

### Antioxidant activity

The antioxidant activity of the milk thistle oil samples was evaluated using the two most common radical scavenging assays DPPH and ABTS. The DPPH test has been widely used for the estimation of the antioxidant capacity of plant and health food extracts due to its simplicity, stability and reproducibility [8]. All the tested oil samples showed strong radical scavenging activity which varied from 70.25 to 96.42% (Table 1). The difference between DPPH and ABTS values obtained for the same sample might be due to the fact that ABTS assay is applicable to both hydropholic and lipopholic antioxidants systems, however DDPH assay is applicable to hydrophobic system [31]. Milk thistle seed oil had a higher DPPH value compared with other vegetables oils such as unheated sesame oil which had DPPH value of 69.2% [35]. This result is in agreement with previous studies that showed a high free radical scavenging capacity for cold-pressed milk thistle oil [31] and ethanolic extracts of milk thistle seeds [10]. Kiralan et al. [41] reported that olive oil was able to quench 52.31–94.91% of DPPH radicals, depending in olive cultivar. Using toluene to dissolve the DPPH and the oil samples, the order of effectiveness of some vegetable oils in inhibiting free radicals was as follows: coriander >black cumin> cottonseed> peanut> sunflower> walnut> hemp seed> linseed >olive >niger seed [42]. Solvent may influence the hydrogen-donating capacity of the antioxidant and affect the antioxidant activity of samples [34, 42].

Our results showed that the antiradical action of oil samples was affected by the seed maturity stage (Table 1). The immature seed oil that contained the highest policosanol amount showed the maximum antiradical activity (96.42% and 90.35% for DPPH test and ABTS assay, respectively). A decline in the antioxidant activity was also observed for carob, pepper and guava throughout fruit ripening [7–9]. The stronger antioxidant capacity of immature seed oil compared to mature seed oil may be due to the differences in their content and composition of unsaponifiable matter. In our previous study, we found that the amount of total unsaponifiable matter decreased during milk thistle seed maturation, while the total lipid content increased [29]. Additionally, Ramadan and Mörsel [42] reported a positive correlation between the radical scavenging activity of vegetable oils and their levels of unsaponifiables.

During seed maturation, a high positive correlation was observed between total policosanol content and antiradical activity of seed oil samples through DPPH (*r* = 0.952) and ABTS (r = 0.899) assays (Table 4). This result suggested that policosanol content may contribute to the antioxidant potential of oil sample. The antioxidant activity was correlated not only with the total amount of antioxidants, but also with the presence of selected compounds [42]. Dabbour et al. [31] reported that the high free radical scavenging capacity of cold-pressed milk thistle seed oil may be due to the presence of phenolic compounds and alpha-tocopherol. For the ethanolic extract of milk thistle seeds, the antioxidant potential was mainly correlated to the total content of flavonoids and phenolics rather than the content of individual compounds including silybin [10]. Other reports indicated a positive correlation between radical scavenging capacity of seed oils and their levels of unsaponifiables and phytosterols, while a negative relation was noted with the amounts of phenolics and tocopherols [42]. Conversely, Kozłowska et al. [34] reported a positive correlation between total phenolics content and antioxidant activity of seed oils and a negative correlation with total sterols. The general consensus within the literature is that the antioxidant capacity appears to be related to the complementary role of different synergic compounds rather than being ascribed to one or a few compounds [10, 42].

### In vitro anti-arthritic activity

Protein denaturation is a process in which proteins lose their tertiary structure and secondary structure. It is one of the causes of arthritic disease and inflammation [37]. Mechanism of denaturation probably involves alteration in electrostatic, hydrogen, hydrophobic and disulphide bonding [36]. In vitro anti-arthritic activity of studied oil samples was evaluated with bovine serum albumin (BSA) denaturation and egg albumin denaturation methods. Our study reveals that the milk thistle oil is capable to inhibit the denaturation of protein (Table 3). The oil extracted from mature seeds showed inhibition of denaturation of BSA and egg albumin by 72.49% and 59.89%, respectively. This result suggested that milk thistle oil might prevent denaturation of protein in rheumatoid arthritis and could be used as a potential anti- arthritic agent. Previously published studies examined the anti-arthritic activity of various plant extracts. The ethanolic extract of *Oryza sativa* inhibited the egg albumin and bovin serum denaturation by 84.15% 60.47%, respectively [36]. The methanolic stem extract of *Cuscuta pedicellata* also exhibited strong inhibition of protein denaturation [43]. Kamble et al. [44] indicated that ethanolic extract of leaves of *Vitex negundo* and Punica granatum showed potential anti-arthritic activity as compared to aqueous extract.

**Table 3:** Effect of milk thistle oil on egg albumin and BSA denaturation, during seed maturation

Maturation stage	% of inhibition^a^	% of inhibition^b^
Immature	59.89 ± 1.4	72.49 ± 1.3
Intermediate	78.46 ± 1.2	85.32 ± 1.0
Mature	86.36 ± 1.5	92.53 ± 1.2

Values were expressed as means ± SD of triplicate experiments

^a^% of inhibition of egg albumin denaturation

^b^% of inhibition of bovine serum albumin (BSA) denaturation

Our results showed that the anti-arthritic activity of oil samples was affected by the seed maturity stage, as shown in Table 3. The maximum inhibition of BSA denaturation (92.53%) and egg albumin denaturation (86.36%) were exhibited by the oil extracted from the immature seeds. Throughout seed maturation, the total policosanol content of milk thistle oil was highly correlated with the inhibition of BSA denaturation (0.774) and egg albumin denaturation (0.901) (Table 4).Therefore, the anti-arthritic activity of oil can be attributed, in part, to the policosanol content. Kumari et al. [37] reported that the anti-arthritic activity of methanolic extract of *Rhizophora mucronata* might be due to the presence of active principles such as polyphenolic content, triterpenoids, alkaloids and flavanoids. Though present in small amounts, the minor constituents of dietary oils may supplement the dietary therapies for rheumatoid arthritis [45].

**Table 4:** Correlation coefficients (r) between policoanol content, antioxidant and anti-arthritic activities of milk thistle oil, during seed maturation

	Correlation coefficient
TPC-DPPH effect	0.952
TPC-ABTS effect	0.899
TPC- % of inhibition of EAD	−0.901
TPC- % of inhibition of BSA	0.774

*TPC* Total Policosanol content

*DPPH* 1,1-diphenyl-2-picrylhydrazyl radical

*ABTS* 2,2′-azino-bis (3-ethyl-benzthiazoline-6-sulfonic acid)

*EAD* egg albumin denaturation

*BSA* bovine serum albumin denaturation

## Conclusions

The results of the present study demonstrated that milk thistle oil exhibited potential antioxidant and anti-arthritic activities. Thus, this oil might prevent denaturation of protein in rheumatoid arthritis and could serve as natural antioxidant and anti-arthritic agents for application in the food industries and pharmaceutic. The immature seed oil that contained the highest policosanol amount showed the maximum anti-arthritic and antioxidant activities as compared to mature seed oil. Policosanol contents in the seed oils may have a great impact on their biological effects. Further studies are needed to explore the medicinal value of milk thistle oil and to elucidate the mechanism of the In-vitro anti-arthritic activity of the seed oil.

## Abbreviations

ABTS: 2,2′-azino-bis (3-ethyl-benzthiazoline-6-sulfonic acid)
BSA: Bovine serum albumin
C_22_-OH: Docosanol
C_23_-OH: Tricosanol
C_24_-OH: Tetracosanol
C_26_ –OH: Hexacosanol
C_27−_OH: Heptacosanol
C_28_-OH: Octacosanol
C_29_-OH: Nonacosanol
C_30_-OH: Triacontanol
C_32_-OH: Dotriacontanol
DPPH: 2,2-diphenyl-1-picrylhydrazyl
GC-MS: Gas chromatography coupled to mass spectrometry
SD: Standard deviation

## References

[1] Murugananthan G, Sudheer KG, Sathya CP, Mohan S (2013). Anti-arthritic and anti-inflammatory constituents from medicinal plants. J Appl Pharm Sci.

[2] Elisha IL, Dzoyem JP, McGaw LJ, Botha FS, Eloff JN (2016). The anti-arthritic, anti-inflammatory, antioxidant activity and relationships with total phenolics and total flavonoids of nine south African plants used traditionally to treat arthritis. BMC Complement Altern Med.

[3] Singh G, Ramey DR, Morfeld D, Shi H, Hatoum HT, Fries JF (1996). Gastrointestinal tract complications of nonsteroidal anti-inflammatory drug treatment in rheumatoid arthritis: a prospective observational cohort study. Arch Intern Med.

[4] Sarafian TA, Kouyoumjian S, Tashkin D, Roth MD (2002). Synergistic cytotoxicity of Delta(9)-tetrahydrocannabinol and butylated hydroxyanisole. Toxicol Lett.

[5] Saito M, Sakagami H, Fujisawa S (2003). Cytotoxicity and apoptosis induction by butylated hydroxyanisole (BHA) and butylated hydroxytoluene (BHT). Anticancer Res.

[6] Gülçin I (2012). Antioxidant activity of food constituents: an overview. Arch Toxicol.

[7] Adedayo BC, Oboh G, Akindahunsi AA (2010). Changes in the total phenol content and antioxidant properties of pepper fruit (*Dennettiatripetala*) with ripening. Afr. J Food Sci.

[8] Benchikh Y, Louaileche H, George B, Merlin A (2014). Changes in bioactive phytochemical content and in vitro antioxidant activity of carob (*Ceratoniasiliqua*L.) as influenced by fruit ripening. Ind Crop Prod.

[9] Gull J, Sultana B, Anwar F, Naseer R, Ashraf M, Ashrafuzzaman M (2012). Variation in antioxidant attributes at three ripening stages of guava (*Psidiumguajava* L.) fruit from different geographical regions of Pakistan. Molecules.

[10] Lucini L, Kane D, Pellizzoni M, Ferrari A, Trevisi E, Ruzickova G (2016). Phenolic profile and in vitro antioxidant power of different milk thistle [*Silybummarianum* (L.) Gaertn.] cultivars. Ind Crop Prod.

[11] Giacometti J (2001). Determination of aliphatic alcohols, squalene, a-tocopherol and sterols in olive oils: direct method involving gas chromatography of the unsaponifiable fraction following silylation. Analyst.

[12] Harrabi S, Mayer MP, Kallel H (2009). Policosanol distribution and accumulation in developing corn kernels. Food Chem.

[13] Montserrat-de la Paz S, García-Giménez M, Ángel-Martín M, Pérez-Camino M, Arche AF (2014). Long-chain fatty alcohols from evening primrose oil inhibit the inflammatory response in murine peritoneal macrophages. J Ethnopharmacol.

[14] Guardamagna O, Abello F, Baracco V, Stasiowska B, Martino F (2011). The treatment of hypercholesterolemic children: efficacy and safety of a combination of red yeast rice extract and policosanols. Nutr Metab Cardiovasc Dis.

[15] Gouni-Berthold I, Berthold HK (2002). Policosanol: clinical pharmacology and therapeutic significance of a new lipid-lowering agent. Am Heart J.

[16] Ham H, Yoon SW, Kim IH, Kwak J, Lee JS, Jeong HS (2015). Protective effects of unsaponifiable matter from rice bran on oxidative damage by modulating antioxidant enzyme activities in HepG2 cells. LWT-food. Sci Technol.

[17] AMd O, Conserva LM, JNdS F, FdA B, RPL L, Barreto E (2012). Antinociceptive and anti-inflammatory effects of octacosanol from the leaves of Sabiceagrisea Var. Grisea in mice. Int J Mol Sci.

[18] Wang T, Liu Y, Yang N, Ji C, Chan P, Zuo P (2012). Anti-parkinsonian effects of octacosanol in 1-methyl-4-phenyl-1, 2, 3, 6 tetrahydropyridine-treated mice. Neural Regener Res.

[19] Warren PR, Burger RA, Sidwell RW, Clark LL (1992). Effect of triacontanol on numbers and functions of cells involved in inflammatory responses. Proc Soc Exp Biol Med.

[20] Ramanarayan K, Bhat A, Shripathi V, Swamy GS, Rao KS (2000). Triacontanol inhibits both enzymatic and nonenzymatic lipid peroxidation. Photochem.

[21] Millán J, Cicero AFG, Torres F, Anguera A (2016). Effects of a nutraceutical combination containing berberine (BRB), policosanol, and red yeast rice (RYR), on lipid profile in hypercholesterolemic patients: a meta-analysis of randomised controlled trials. Clín InvestigaciónenArteriosclerosis.

[22] Choi SJ, Park SY, Park JS, Park SK, Jung MY (2016). Contents and compositions of policosanols in green tea (*Camellia sinensis*) leaves. Food Chem.

[23] Jung DM, Lee MJ, Yoon SH, Jung MY (2011). A gas chromatography-tandem quadrupole mass spectrometric analysis of policosanols in commercial vegetable oils. J Food Sci.

[24] Hwang KT, Weller CL, Cuppett SL, Hannan MA (2004). Policosanol contents and composition of grain sorghum kernels and dried distillers grains. Cereal Chem.

[25] Laguna A, Magraner J, Carbajal D, Arruzazabala ML, Más R, García M (1997). A mixture of higher primary aliphatic alcohols, its obtention from sugar cane wax and its pharmacological uses.US 5663156 a.

[26] Harrabi S, Romdhane H, Daassa M, Fellah H (2015). Fatty acid and triacylglycerol compositions of milk thistle seeds growing wild in Tunisia (*Silybummarianum* L.). ActaAlimentaria.

[27] Růžičková G, Fojtová J, Součková M (2011). The yield and quality of milk thistle (*Silybummarianum* (L). Gaertn.) seed oil from the perspective of environment and genotype – a pilot study. ActaFytotechnicaetZootechnica.

[28] Bahl JR, Bansal RP, Goel R, Kumar S (2015). Properties of the seed oil of a dwarf cultivar of the pharmaceutical silymarin producing plant silybummarianum (L.) gaertn. Developed in India. I J Nat Prod Res.

[29] Harrabi S, Curtis S, Hayet F, Mayer PM (2016). Changes in the sterol compositions of milk thistle oil (*Silybiummarianum* L.) during seed maturation. Grasas Aceites.

[30] Fathi-Achachlouei B, Azadmard-Damirchi S (2009). Milk thistle seed oil constituents from different varieties grown in Iran. J Am Oil Chem Soc.

[31] Dabbour IR, Al-Ismail KM, Takruri HR, Azzeh FS (2014). Chemical characteristics and antioxidant content properties of cold pressed seed oil of wild milk thistle plant grown in Jordan. Pak J Nutr.

[32] Hasanloo T, Bahmanei M, Sepehrifar R, Kalantari F (2008). Determination of tocopherols and fatty acids in seeds of Silybummarianum (L.) Gaerth. J Med Plants.

[33] Folch J, Lees M, SGM S (1957). A simple method for the isolation and purification of total lipids from animal tissues. J Biol Chem.

[34] Kozłowska M, Gruczyńska E, Ścibisz I, Rudzińska M (2016). Fatty acids and sterols composition, and antioxidant activity of oils extracted from plant seeds. Food Chem.

[35] Rubalya VS, Neelamegam P (2015). Selective ABTS and DPPH- radical scavenging activity of peroxide from vegetable oils. Int Food Res J.

[36] Rahman H, Eswaraiah MC, Dutta AM (2015). In-vitro anti-inflammatory and anti-arthritic activity of Oryza Sativa Var. joha rice (an aromatic indigenous rice of Assam). Am Eurasian J Agric Environ Sci.

[37] Kumari CS, Yasmin N, Hussain MR, Babuselvam M (2015). *In vitro* anti-inflammatory and anti-arthritic property of *rhizoporamucronata* leaves. Int J Pharma Sci Res.

[38] Gülz PG, Müller E, Prasad BN (1991). Developmental and seasonal variations in the epicuticular waxes of tiliatomentosa leaves. Phytochemistry.

[39] Irmak S, Jonnal RS, MacRitchie F (2008). Effect of genetic variation on phenolic acid and policosanol contents of Pegaso wheat lines. J Cereal Sci.

[40] Naeem M, Khan MMA (2012). Moinuddin. Triacontanol: a potent plant growth regulator in agriculture. J Plant Interac.

[41] Kıralan M, Bayrak A, Ozkaya MT (2009). Oxidation stability of virgin olive oils from some important cultivars in East Mediterranean area in Turkey. J Am Oil Chem Soc.

[42] Ramadan MF, Mörsel JT (2006). Screening of the antiradical action of vegetable oils. J Food Compos Anal.

[43] Naz R, Ayub H, Nawaz S, Islam ZU, Yasmin T, Bano A, Wakeel A, Zia S, Roberts TH (2017). Antimicrobial activity, toxicity and antiinflammatory potential of methanolic extracts of four ethnomedicinal plant species from Punjab, Pakistan. BMC Complement Altern Med.

[44] Kamble AA, Khan ND, Khan ZH, Mular SM, Sohail S (2017). In vitro anti-arthritic activity of vitexnegundo and punicagranatum. Res J Pharm Sci.

[45] Yadav NV, Sadashivaiah, Ramaiyan B, Acharya P, Belur L, Talahalli RR (2016). Sesame oil and Rice bran oil ameliorates adjuvant-induced arthritis in rats: distinguishing the role of minor components and fatty acids. Lipids.

